# Real-Time Fatigue Detection Algorithms Using Machine Learning for Yawning and Eye State

**DOI:** 10.3390/s24237810

**Published:** 2024-12-06

**Authors:** Fazliddin Makhmudov, Dilmurod Turimov, Munis Xamidov, Fayzullo Nazarov, Young-Im Cho

**Affiliations:** 1 Department of Computer Engineering, Gachon University, Seongnam 1342, Republic of Korea; fazliddin12@gachon.ac.kr (F.M.); dilmurod@gachon.ac.kr (D.T.); 2 Department of Artificial Intelligence and Information Systems, Samarkand State University, Samarkand 140104, Uzbekistan; samsungmunis@gmail.com (M.X.); fayzulla-samsu@mail.ru (F.N.)

**Keywords:** drowsiness detection, convolutional neural networks (CNNs), VGG16, yawning detection, eye closure detection, Haar cascade classifier, deep learning, facial feature analysis

## Abstract

Drowsiness while driving is a major factor contributing to traffic accidents, resulting in reduced cognitive performance and increased risk. This article gives a complete analysis of a real-time, non-intrusive sleepiness detection system based on convolutional neural networks (CNNs). The device analyses video data recorded from an in-vehicle camera to monitor drivers’ facial expressions and detect fatigue indicators such as yawning and eye states. The system is built on a strong architecture and was trained using a diversified dataset under varying lighting circumstances and facial angles. It uses Haar cascade classifiers for facial area extraction and advanced image processing algorithms for fatigue diagnosis. The results demonstrate that the system obtained a 96.54% testing accuracy, demonstrating the efficiency of using behavioural indicators such as yawning frequency and eye state detection to improve performance. The findings show that CNN-based architectures can address major public safety concerns, such as minimizing accidents caused by drowsy driving. This study not only emphasizes the need of deep learning in establishing dependable and practical driver monitoring systems, but it also lays the groundwork for future improvements, such as the incorporation of new behavioural and physiological measurements. The suggested solution is a big step towards increasing road safety and reducing the risks associated with driver weariness.

## 1. Introduction

Drowsiness behind the wheel is a major factor contributing to road accidents, posing a serious threat to drivers and others on the road. A lack of sleep affects cognitive abilities, slowing reaction times, impairing decision-making, and significantly increasing the risk of accidents. Research has shown that the effects of drowsiness on driving performance can be as dangerous as those caused by alcohol consumption. Data from the National Highway Traffic Safety Administration (NHTSA) highlight the gravity of this issue, with drowsy driving accounting for an estimated 72,000 crashes, 44,000 injuries, and 800 fatalities annually in the U.S. alone [1]. Furthermore, a considerable number of drivers admit to falling asleep while driving at least once, underscoring the pressing need for efficient drowsiness detection systems. Recent developments in deep learning and computer vision have provided new opportunities to combat driver fatigue. Convolutional neural networks (CNNs), recognized for their high accuracy in image classification tasks, are showing promise in detecting drowsiness-related behaviours, such as eye closure and yawning [2]. Traditional methods for monitoring driver fatigue often involved intrusive techniques, like electroencephalograms (EEGs) and electrocardiograms (ECGs), which are impractical in real-world scenarios due to their discomfort and complexity [3]. As a result, non-invasive detection systems that rely on behavioural cues have garnered increased attention [4]. This study aims to develop a real-time, non-intrusive drowsiness detection system using CNNs to track facial features and alert drivers when signs of fatigue are detected [5]. Our system is designed to use an in-vehicle camera to continuously capture video footage of the driver, with a CNN model analysing the images to identify signs of yawning and measure eye blink frequency—both reliable indicators of drowsiness. Yawning, typically marked by a wide mouth opening, and eye states are recognized symptoms of fatigue. By combining these two factors, the system aims to enhance detection accuracy while minimizing false alarms, thereby promoting safer driving. The research methodology includes crucial steps such as data normalization and preparation. We utilized publicly available facial image datasets labelled for yawning and eye states (open or closed), with images captured in different lighting conditions and angles to improve model robustness. Preprocessing was carried out using Haar cascade classifiers to detect facial regions, which were then resized for input into the CNN. In the following sections, we detail the review of related works in this field and our methodology, covering the dataset structure, model architecture, training process, and evaluation metrics. This work underscores the potential of deep learning-based approaches to address the critical issue of drowsiness detection, contributing to enhanced road safety applications.

## 2. Related Work

Over the past few years, significant advancements have been made in the field of yawning detection, particularly with the integration of machine learning and computer vision technologies, which have enabled real-time detection in various scenarios. Early efforts, such as the work by Akrout et al., utilized the Viola–Jones algorithm for mouth and face localization in video frames, with AdaBoost for classification. By segmenting lips and applying active contour techniques for edge detection, they provided an approach that focused on mouth shape recognition to detect yawning via the Zero-Crossing method [6]. Abtahi et al. presented a more lightweight computational approach, developing an embedded smart camera system to monitor drivers for signs of yawning. This work emphasized the use of the modified Viola–Jones algorithm and back-projection methods to assess mouth movements during yawning. The system was designed for driver drowsiness detection, monitoring other fatigue indicators such as eye closure and head movements [7]. Further developments in 2017 by Zhang et al. introduced a deep learning-based solution using a combination of convolutional neural networks (CNNs) and Long Short-Term Memory (LSTM) models. Their approach applied the GoogleNet architecture to extract yawning-specific features from video frames, showcasing how CNNs can be employed for video-based yawning detection with sequential video input [8]. In 2018, Mane et al. leveraged TensorFlow to create an open-source framework for object detection and tracking using CNNs. Their work addressed challenges in detecting objects in complex environments, although issues like drastic changes in the scene and target drift remained unsolved. Despite these challenges, their approach demonstrated the applicability of deep learning in real-time yawning detection [9]. Raizal et al. enhanced yawning detection by introducing mouth and eye classifiers based on OpenCV and the Viola–Jones algorithm. They introduced metrics like the Eye Aspect Ratio (EAR) and Mouth Aspect Ratio (MAR) to monitor yawning and eye closure, which provided a more reliable way to detect driver fatigue [10]. Meanwhile, Sukjamsri et al. incorporated support vector machines (SVMs) for feature classification, specifically focusing on detecting mouth states in front-facing camera setups. Yang et al. (2020) proposed a more advanced system using a Receptive Field Enhanced Multi-Task Cascaded CNN (RFE-MTCNN) model. This system, although initially hindered by low precision in detecting smaller facial targets, enhanced the generalization capacity of yawning detection systems by expanding the receptive field through novel CNN structures [11]. Another interesting approach by Wu et al. combined three factors—eye blinking, yawning, and body posture—for a hybrid drowsiness detection system. Using advanced image processing techniques and the HALCON system, they were able to improve yawning discrimination accuracy based on facial region statistics [12]. Lastly, Hu et al. introduced “DrowsyDet”, a mobile application for detecting drowsiness through facial data analysis. This system combined CNN-based models for eyes, mouth, and overall facial states, and though it lacked real-time precision, it presented a promising non-intrusive solution [13]. These studies demonstrate a clear trend towards the integration of machine learning and deep learning methods for real-time yawning detection. The use of CNNs, LSTMs, and support vector machines (SVMs) has been pivotal in enhancing system accuracy, while frameworks like OpenCV and TensorFlow have provided researchers with robust tools for implementing these models. The evolution of these approaches marks a significant step towards more reliable and efficient driver monitoring systems.

Over the years, many approaches have been proposed to detect driver drowsiness using image-based and physiological metrics. Traditional algorithms, such as the Viola–Jones method and SVM classifiers, have shown reasonable success in specific contexts. However, these methods often struggle to generalize effectively under diverse real-world conditions, such as varying lighting and camera angles. For instance, while studies [6,7] implemented lightweight computational techniques using modified Viola–Jones and AdaBoost, their effectiveness was limited by the lack of robustness in feature selection processes under complex scenarios.

Subsequent methods leveraging deep learning frameworks, such as the GoogleNet-LSTM combination [8], addressed some limitations by incorporating temporal dependencies. However, these architectures often require extensive computational resources, making them less feasible for real-time applications. The rise of CNN-based methods, as demonstrated in the works of [9,12], has shown improved accuracy due to their ability to extract robust features. Nonetheless, a common challenge among these studies remains, namely achieving a balance between computational efficiency and detection accuracy.

## 3. Methodology

**Figure 1:**
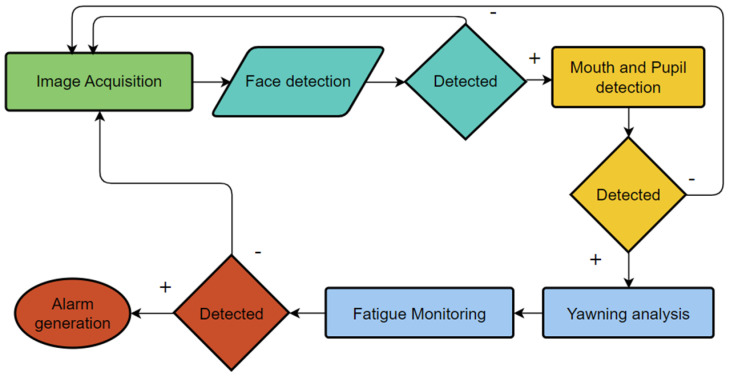
Process of drowsiness detection system.

When individuals experience mental fatigue, they may become drowsy and lose focus on their tasks. While fatigue itself is not a disease, it can be mitigated by rest and sleep. However, this can be particularly challenging when operating vehicles that require constant vigilance, such as trains, buses, or cars. The lack of attention in these scenarios can lead to serious accidents. Consequently, numerous studies have focused on exploring the physiological mechanisms of human drowsiness and evaluating fatigue levels. Determining the exact level of fatigue is complex, as various factors can influence it in different ways [14]. To effectively monitor fatigue, a comprehensive system is necessary—one that collects data from multiple sources and integrates these data to provide a clear and accurate depiction of a person‘s state. The process of how this kind of fatigue detection system can be organised is shown in Figure 1 [15].

To decide whether a person is drowsy or not, measurement metrics or indicators are needed. These indicators play a crucial role in assessing fatigue and drowsiness levels. Common metrics include the following:

1. **Eye State**: Monitoring whether the eyes are open or closed can provide a direct indicator of drowsiness. Eye state is a strong signal of sleep onset.
2. **Yawning**: The frequency and intensity of yawns can indicate levels of tiredness, as yawning often increases when a person is fatigued.
3. **Head Movement**: Unusual head movements, such as nodding or sudden jerks, can signal a loss of attention or imminent sleep.
4. **Electroencephalogram (EEG)**: Measuring brainwave activity provides insights into a person’s mental state. Specific patterns, such as increased theta waves, are associated with drowsiness.
5. **Other Physiological Metrics**: Additional indicators like heart rate variability (HRV), skin conductance, and eye blink frequency can also be used to assess fatigue levels [16].

These metrics, which can be collected through various sensors and analysed using advanced algorithms, enable the system to detect signs of drowsiness with high accuracy. Such systems are vital for enhancing safety in situations that require sustained attention and quick reactions. In most cases, researchers are focused on image processing, which is becoming more effective and more accurate in terms of results. In Table 1, some image-based measures are described [17].

The features described above are efficient in particular cases; however, it is suggested to combine these measures when the model is being built. Table 2 shows the statistic about the accuracy of each feature and the used classification methods [18].

Our drowsiness detection system is designed to identify yawning and eye states as two combined features using a convolutional neural network (CNN). This section details the various steps and components involved in the development and implementation of the system, including data normalisation, preprocessing, model architecture, training, and real-time detection. The core of our system is a convolutional neural network (CNN) designed to classify the input images into the predefined categories. The CNN architecture consists of several layers, each serving a specific purpose:

1. **Convolutional Layers:**

Separate convolutional layers are used to identify eye state and yawning, extracting characteristics such as forms, edges, and movement patterns. These layers concentrate on capturing the distinctive properties of each indicator. Eye State Pipeline: convolutional filters detect patterns indicating whether the eyes are open or closed. Yawning Detection Pipeline: filters focus on identifying the mouth region’s shape and movement to determine if a person is yawning. Both pipelines are implemented as separate branches within the same network.

(1) Conv(x)=x*w+b,

where * denotes the convolution operation, *x* is the input image, *w* is the filter, and *b* is the bias term.

2. **Activation Function (ReLU):**

The Rectified Linear Unit (ReLU) activation function introduces non-linearity into the model, enabling it to learn complex patterns in eye state and yawning features; ReLU is applied after each convolutional operation [19].

(2) ReLU(x)=max(0,x),

3. **Pooling Layers:**

Max pooling layers reduce the spatial dimensions of the feature maps, retaining the most significant features. This procedure reduces the feature maps, increasing computing efficiency and decreasing overfitting.

(3) $$MaxPool(x)=\max(\{x_{i,j}:(i,j)\in pool\;region\}),$$

4. **Feature Fusion:**

Features extracted from the eye state pipeline and yawning pipeline are concatenated into a single-feature vector and this fusion combines eye and yawning features, allowing the network to consider both indicators simultaneously.

5. **Fully Connected Layers:**

The fused feature vector is sent through dense layers to learn high-level representations and categorize the input (yawn, no yawn, eyes closed, eyes open).

(4) FC(x)=Wx+b,

6. **Dropout Layer:**

Dropout is applied to prevent overfitting by randomly setting a fraction of input units to zero during training.

(5) Dropout(x)=x·Bernoulli(p),

7. **Output Layer:**

The final layer uses a SoftMax activation function to output a probability distribution over the four classes [20].

(6) $$Soft\max(x_{i})=\frac{e^{x_{i}}}{\sum_{j}e^{x_{j}}},$$

The model was trained using the labelled dataset with the following parameters:

- Loss Function: Categorical Cross-Entropy;
- Optimizer: Adam optimizer, chosen for its efficiency in handling large datasets and adaptive learning rate;
- Epochs and Batch Size: the model was trained for 25 epochs with a batch size of 32.

**Figure 2:**
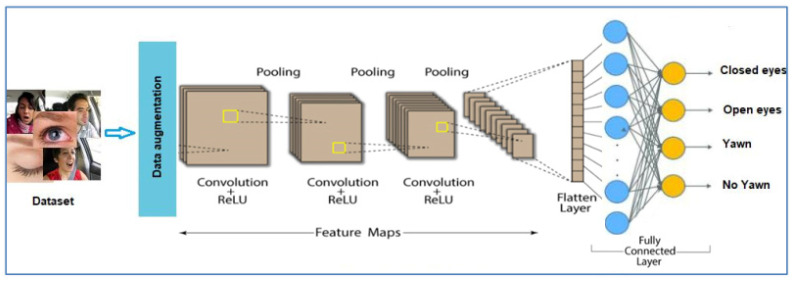
The architecture of the CNN model.

The training process involved splitting the dataset into training and validation sets to evaluate the model‘s performance and prevent overfitting. The model parameters were adjusted to minimize the loss function, ensuring accurate classification of the input images. The model architecture can be identified in Figure 2.

### 3.1. Dataset Description

**Figure 3:**
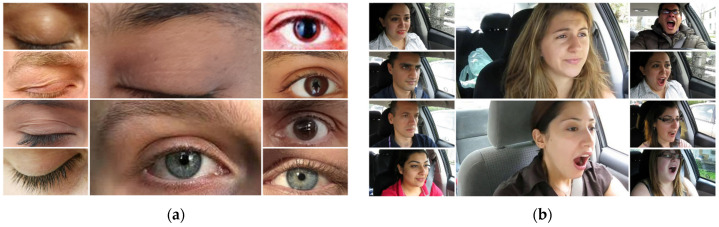
Samples of dataset for yawning and eye state: (**a**) samples from MRL eye dataset; (**b**) samples from YawDD dataset.

In our study, we used two publicly available datasets, YawDD [21] and MRL Eye [22], which contain a total of 2900 images. The YawDD dataset consists of 349 videos totalling 5 GB in size, making analysis difficult. That is why we extracted 1450 frames from these videos for the yawning dataset and another 1450 images from the MRL Eye dataset for eye state classification. The initial step was to gather a comprehensive dataset of facial images, categorized into four different states: “Closed”, “Open”, “Yawning”, and “No Yawning”. In Figure 3 below, an example of our dataset is shown.

### 3.2. Data Preprocessing

Python is widely recognized as a leading language for machine learning due to its simplicity, clear syntax, and powerful capabilities for text manipulation and processing. It was chosen for this prototype because it supports the implementation of abstract concepts without requiring extensive low-level programming [23]. The libraries used in our project are as follows:

- OpenCV: used for real-time computer vision, accessing the camera, and utilizing Haar feature-based classifiers for object detection.
- TensorFlow: essential for large-scale machine learning algorithms and numerical computation; it is also a prerequisite for Keras.
- NumPy: crucial for creating and handling arrays in Python.
- Pandas: used for data manipulation and analysis, providing data structures and operations for efficiently handling structured data.
- Imutils: primarily used for image preprocessing tasks like resizing, translation, rotation, and skeletonization.
- Keras: a high-level neural network library used for developing classification models.

### 3.3. Haar Cascade Classifier

OpenCV provides various tools for attribute detection, including Haar cascade classifiers, which are used for object detection in images and videos. This algorithm operates through four stages:

1. Haar Feature Selection: this involves selecting adjacent rectangular regions in a detection window.
2. Creating Integral Images: this efficiently calculates the sum of pixel values in a rectangular subset.
3. Adaboost Training: this selects the best features and trains the classifier.
4. Cascading Classifiers: these combine multiple stages, each with weak learners, to form a strong classifier.

These classifiers are used in the prototype to detect facial and eye landmarks in images and real-time scenarios, ensuring accurate attribute detection during data preprocessing [24]. We used Haar cascade classifiers to detect faces and eyes in the images. The classifiers were applied to each image to identify regions containing faces and eyes, which were then extracted for further processing. When it comes to eye detection, we used the Eye Aspect Ratio technique (EAR), which is a simple yet effective metric used for eye blink detection and eye state classification (open or closed). It is widely used in various computer vision applications, such as drowsiness detection systems. The EAR is based on the ratio of distances between facial landmarks around the eyes. It remains approximately constant when the eye is open but decreases when the eye is closed. By monitoring this ratio over time, one can detect blinks or eye state, which can indicate drowsiness. The EAR is calculated using the vertical distances between pairs of points and the horizontal distance between the two endpoints. For example, for the left eye, the formula is as follows:

(7) $$\mathrm{EAR}=\frac{\left\|P2-P6\right\|+\left\|P3-P5\right\|}{2\left\|P1-P4\right\|},$$

where P1, P2, P3, P4, P5, and P6 are the 2D coordinates of the eye landmarks and ∥·∥ denotes the Euclidean distance between two points [25].

## 4. Results

**Figure 4:**
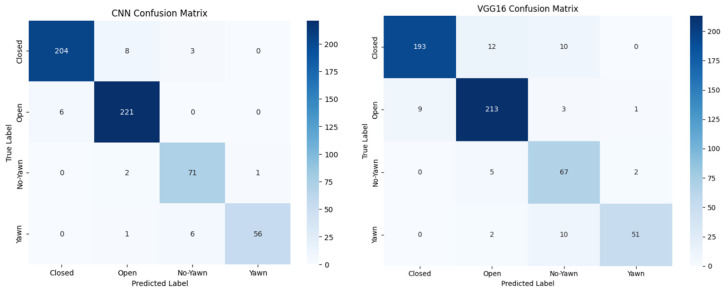
Confusion matrices of CNN and VGG16 models.

The proposed drowsiness detection system leverages image-based metrics and a convolutional neural network (CNN) to classify eye states and yawning. The comprehensive system undergoes rigorous evaluation using two publicly available datasets, YawDD and MRL Eye, consisting of a total of 2900 images. In total, 70% of the dataset is utilized for training, with the remaining 30% used for testing. The dataset utilized in the training phase differs from that used in the testing phase. Data augmentation techniques are applied to the training set to address picture alterations and noise. Geometric transformations, such as zooming, flipping, and rotating, are used to enhance data and provide new results. New data are generated during the learning process. All data are transmitted using forward–backward propagation. During each iteration, the image goes via a data augmentation layer before reaching the convolution layers of the DL model. Finally, the model improves the accuracy and reduces errors with each iteration. The following subsections outline the results obtained from this evaluation, focusing on accuracy, loss metrics, confusion matrix analysis, and performance metrics. Table 3 below shows how the two different models were compared considering several aspects such as the number of layers, epochs, and spent time. The confusion matrices (Figure 4) provide a detailed breakdown of the system’s classification performance based on the two models across the four classes: “Closed”, “Open”, “Yawning”, and “No Yawning”. Matrices reveal that the system achieved high accuracy in distinguishing between these classes, with most errors occurring in the classification of “yawn” and “no_yawn” states. This misclassification can be attributed to the subtle facial differences in these states, which may not be as pronounced as those between “Closed” and “Open” eyes.

The models were created in Python using Google Collab and supported by OpenCV, Keras, and Tensorflow libraries on a PC with the following configuration: Intel^®^ Core i5 13th generation CPU, 8 GB of RAM, Windows 11 64-bit, and a web camera. The total number of epochs ranged from 15 to 25 depending on the model. As a result, the processing duration varied amongst models. It increased unless the number of layers grew, and our proposed system trained a single image for each model in an average time of 0.31 s.

**Figure 5:**
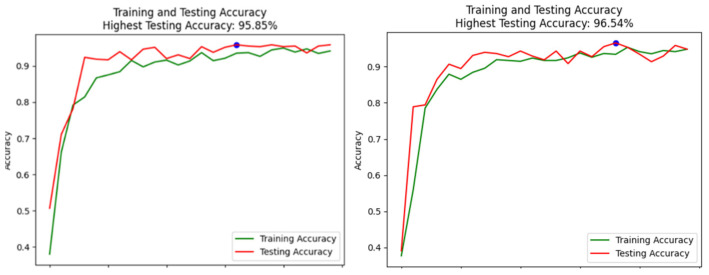
Training and testing accuracy. The blue dot indicates the epoch where the highest testing accuracy was achieved.

Specifically, the system displayed the highest accuracy in detecting “Open” eyes, with minimal false positives for the other states. The correct detection of “Closed” eyes was also high, underscoring the system’s effectiveness in identifying potential drowsiness. The overall accuracy across all classes indicates a well-balanced model capable of handling the variability within the dataset. The training and testing accuracy curves and results (Figure 5) demonstrate the models learning progression over 25 and 10 epochs. The VGG16 model achieved a peak testing accuracy of 95.85% and the traditional CNN achieved 96.54%. Notably, both training and testing accuracies exhibit a consistent upward trend, with the testing accuracy closely following the training accuracy. This pattern indicates minimal overfitting, suggesting that the model generalizes well to unseen data.

The gradual convergence of the accuracy curves reflects the model’s ability to learn complex patterns in the data effectively. The slight variations observed towards the later epochs may be attributed to the inherent complexity of distinguishing between similar facial expressions, such as mild yawning and non-yawning states.

**Figure 6:**
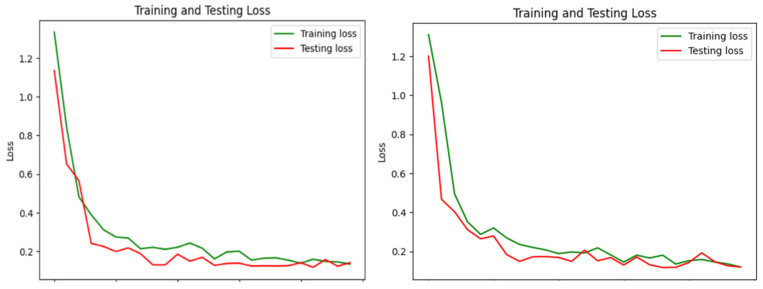
Training and testing loss.

The loss curves for both training and testing datasets (Figure 6) show a significant decrease over the epochs, with the final loss values stabilizing at low levels. The initial steep decline in loss values highlights the model’s rapid learning phase, where it quickly identifies key features in the data. As training progresses, the loss values for both datasets continue to decrease, albeit at a slower rate, indicating a gradual refinement of the model’s predictions.

The final testing loss remains slightly higher than the training loss, suggesting a small degree of overfitting. However, this difference is not substantial enough to adversely impact the model’s generalization capability. The low loss values towards the end of the training phase confirm the model’s robustness and reliability in classifying the input images accurately. Table 4 and Table 5 include essential performance metrics—precision, recall, F1-score, and support—for assessing the proposed drowsiness detection system in four categories: yawn, no_yawn, closed, and open. These metrics provide a thorough understanding of the model’s classification performance and capacity to generalize to new data.

In a comparison of the two models for facial emotion recognition, the CNN outperformed VGG16 across multiple evaluation parameters. The results, as stated in the confusion matrices and classification reports, show that the CNN model outperforms VGG16 in terms of accuracy, precision, recall, and F1-score. The CNN model had higher precision, which means it was more accurate at selecting the proper class with fewer false positives. It also had a stronger recall, correctly detecting the majority of the target facial expressions while lowering false negatives. The F1-score of the CNN model, which balances precision and recall, was significantly higher, indicating a better overall trade-off between these two criteria. On the other hand, the VGG16 model performed satisfactorily but had lower precision and recall values, resulting in a lower F1-score. This shows that the VGG16 model suffered with misclassifications and missed a bigger proportion of real positive cases, resulting in a lower overall performance compared to the CNN model. The support, or the number of samples per class, was identical for both models. To summarize, the CNN model surpasses VGG16 in the task of facial expression recognition, most likely due to its architecture, which is better adapted to capturing the complex patterns and minor changes present in facial emotions. The CNN model’s enhanced performance indicates that it is more adept at correctly recognizing and categorizing facial expressions, which makes it a more dependable option for this task. Future studies could concentrate on improving the CNN model’s accuracy and resilience, which could make it even more useful for tasks involving the recognition of facial expressions in practical settings.

### Real-Time Detection

**Figure 7:**
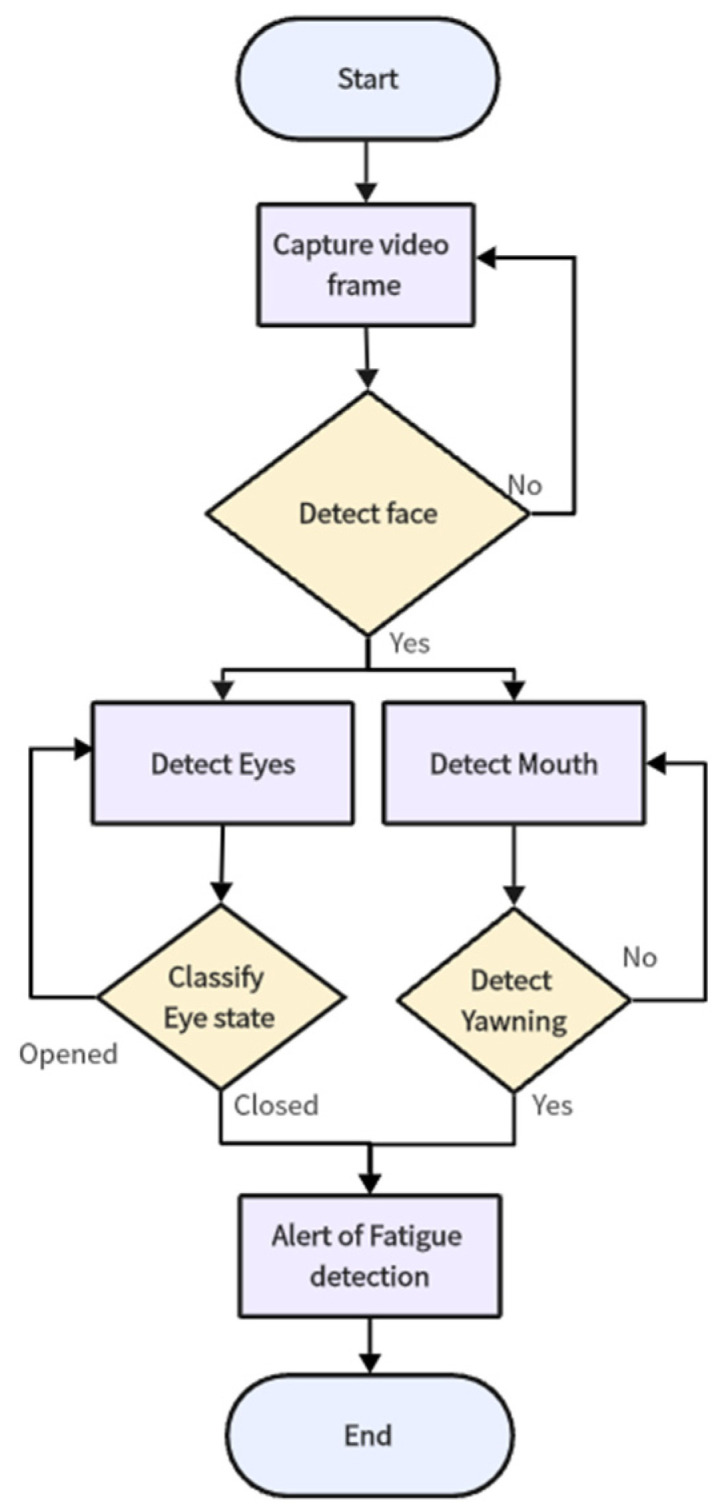
Flow chart for proposed algorithm.

The real-time detection system integrates the trained CNN model with a live video feed from a webcam. The steps of the proposed algorithm for our fatigue detection system are described as follows and the flow chart of the algorithm is shown in Figure 7:

1. Capture Video Frame: the webcam captures continuous video frames of the driver.
2. Face and Eye Detection: each frame is processed using the Haar cascade classifiers to detect faces and eyes.
3. Preprocessing: the detected facial regions are resized and normalized to match the input size expected by the CNN.
4. Prediction: the preprocessed images are passed through the CNN model to predict the state (yawning, not yawning, eyes open, eyes closed).
5. Alert Mechanism: if the model detects signs of drowsiness (eye state or frequent yawning), an alarm is triggered to alert the driver.

**Figure 8:**
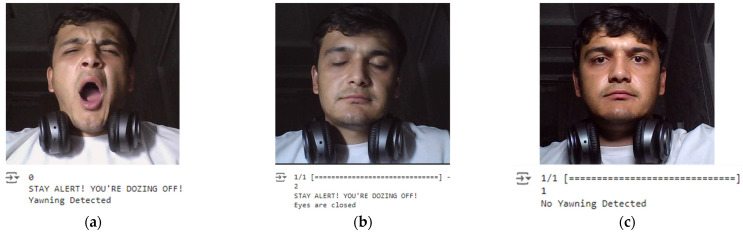
Real-time drowsiness detection example: (**a**) sample where yawning is detected; (**b**) sample where closed eyes are detected; (**c**) sample where neither yawning nor eye state are detected.

This methodology ensures a robust and efficient system for real-time drowsiness detection, leveraging advanced deep learning techniques to enhance road safety. Some examples of real-time drowsiness detection are shown in Figure 8. In real-time testing scenarios, the system demonstrated a quick and accurate response in detecting drowsiness indicators. The combination of Haar cascade classifiers for initial face and eye detection, followed by EAR (Eye Aspect Ratio) calculations, proved effective in identifying eye states. Similarly, the CNN-based approach for yawning detection showed promising results, accurately distinguishing between “Yawning” and “No Yawning” states in real-time.

The integration of these methods enabled the system to function seamlessly, providing a reliable and efficient solution for real-time drowsiness detection. The use of Python and relevant libraries such as OpenCV, TensorFlow, and Keras facilitated the development of a robust system capable of handling various challenges associated with real-time image processing and classification. The proposed system’s accuracy surpasses that of several existing methods that rely on single-feature-based metrics. For example, the Eye Aspect Ratio (EAR) achieved an accuracy of 94.9% in a separate study, whereas our system, which combines multiple features, reached a testing accuracy of 96.54%. This improvement highlights the advantage of incorporating multiple metrics, such as eye state and yawning, to enhance the detection accuracy. Furthermore, the use of CNNs for feature extraction and classification has proven to be more effective than traditional machine learning methods, such as support vector machines (SVMs) and K-Nearest Neighbours (KNNs), especially in handling the complexities of real-time drowsiness detection.

## 5. Conclusions

This study emphasizes the potential of convolutional neural networks (CNNs) in important applications by demonstrating how well they perform hard tasks like sleepiness detection and facial expression identification. In facial expression recognition, the CNN model performed better than VGG16, exhibiting higher accuracy, precision, recall, and F1-score values. Its strong design achieved better performance metrics and guaranteed more accurate classifications by successfully capturing minute details in face expressions. Similar to this, the CNN-based method in the sleepiness detection system demonstrated its capacity to accurately identify fatigue symptoms including yawning and eye states in real time and in a variety of settings, with a testing accuracy of 96.54%. The employment of a non-intrusive method in the drowsiness detection system, paired with a large dataset including diverse lighting and facial angles, emphasizes the model’s usefulness and robustness. The addition of yawning and eye state recognition improved its accuracy, presenting a promising option for real-world applications such as driver monitoring. Both research results highlight CNN-based architectures’ versatility and promise for enhancing safety and addressing public health issues, such as driver fatigue-related traffic accidents. These discoveries pave the way for future developments, such as including more behavioural or physiological data to improve system performance even further. As technology advances, the constant improvement and integration of such systems into commercial applications may greatly enhance safety results, whether by lowering drowsy driving incidents or improving human–computer interaction via facial expression detection. This study demonstrates how deep learning may address real-world difficulties and lays the groundwork for future improvements in public safety and well-being.

Future work can explore advanced techniques like vision transformers (ViTs) for enhanced feature extraction and multimodal approaches combining facial and speech features to improve drowsiness detection [26,27,28]. Additionally, real-time data augmentation methods, such as histogram equalization, could address environmental challenges, further advancing non-intrusive driver monitoring systems.

## Figures and Tables

**Table 1:** Image-based fatigue detection measures.

Features	Description	Year
Blink frequency	This metric tracks the number of times a person’s eyes close within a specific time frame. It helps in identifying patterns that may indicate drowsiness.	2019
Maximum time of eye closure	This measures the maximum duration for which the eyes remain closed. Detecting extended eye closures is critical, as it can signal a high risk of falling asleep, which is especially dangerous for drivers.	2019
Percentage of eyelid closure (PERCLOS)	This metric calculates the percentage of time per minute that the eyes are at least 80% closed. It provides a continuous measure of eye closure over time, indicating the level of drowsiness.	2012
Eye Aspect Ratio (EAR)	The EAR is an indicator of eye openness. The EAR value approaches zero when the eyes are fully closed, while it remains relatively constant when the eyes are open. This measure helps in determining the degree of eye closure at any given moment, allowing for real-time drowsiness detection.	2022
Yawning frequency	This tracks the number of times the mouth opens within a given period. Frequent mouth opening can be a sign of yawning, which is often associated with tiredness.	2021
Head pose	This metric quantifies the movement of a driver’s head by analysing video segments for significant deviations from the normal positions of the three Euler angles (pitch, yaw, and roll). The measurements capture head motions like nodding, shaking, and tilting, which may indicate fatigue or loss of focus.	2003

**Table 2:** Single-feature-based fatigue detection system accuracy.

Image and Video Parameters	Classification Methods	Accuracy	Year
Face	RNN, CNN	60%	2022
Eye	Multilayer perceptron and SVM	94.9	2020
Respiration	SVM and KNN	90% and 83%	2020
Facial features	3D CNN	73.9	2020
Mouth	SVM and Viola–Jones algorithm	75%	2016

**Table 3:** Comparison of two models.

Model	CNN	VGG16
**Number of layers**	5	16
**Number of epochs**	25	10
**Processing time (s)**	940	220
**Accuracy (%)**	96.54	95.85

**Table 4:** Performance metrics for CNN model.

Class	Precision	Recall	F1-Score	Support
Yawn	0.90	0.88	0.89	63
No-Yawn	0.94	0.96	0.95	74
Closed	0.97	0.95	0.96	215
Open	0.97	0.96	0.97	226

**Table 5:** Performance metrics for VGG16 model.

Class	Precision	Recall	F1-Score	Support
Yawn	0.80	0.75	0.77	63
No-Yawn	0.90	0.91	0.90	74
Closed	0.92	0.90	0.91	215
Open	0.93	0.94	0.93	226

## Data Availability

The link for the YawDD dataset is https://www.kaggle.com/datasets/enider/yawdd-dataset, and for the MRL eye dataset, the link is https://www.kaggle.com/datasets/imadeddinedjerarda/mrl-eye-dataset.
